# BatAlign: an incremental method for accurate alignment of sequencing reads

**DOI:** 10.1093/nar/gkv533

**Published:** 2015-07-13

**Authors:** Jing-Quan Lim, Chandana Tennakoon, Peiyong Guan, Wing-Kin Sung

**Affiliations:** 1 Department of Computer Science, National University of Singapore, Singapore 117417; 2 Laboratory of Cancer Epigenome, Division of Medical Sciences, National Cancer Centre Singapore, Singapore 169610; 3 NUS Graduate School for Integrative Sciences and Engineering, (CeLS), #05-01, 28 Medical Drive, Singapore 117456; 4 Department of Computational and Systems Biology, Genome Institute of Singapore, Singapore 138672; 5 UAE University, PO Box 17551, Al Ain, UAE

## Abstract

Structural variations (SVs) play a crucial role in genetic diversity. However, the alignments of reads near/across SVs are made inaccurate by the presence of polymorphisms. BatAlign is an algorithm that integrated two strategies called ‘Reverse-Alignment’ and ‘Deep-Scan’ to improve the accuracy of read-alignment. In our experiments, BatAlign was able to obtain the highest F-measures in read-alignments on mismatch-aberrant, indel-aberrant, concordantly/discordantly paired and SV-spanning data sets. On real data, the alignments of BatAlign were able to recover 4.3% more PCR-validated SVs with 73.3% less callings. These suggest BatAlign to be effective in detecting SVs and other polymorphic-variants accurately using high-throughput data. BatAlign is publicly available at https://goo.gl/a6phxB.

## INTRODUCTION

Aligning sequencing reads to a reference genome is usually the first step in most of the genomic analysis. However, it is harder to align sequencing reads that span across genomic variations back onto a reference genome as the whole-reads do not represent the reference genome exactly. As such, the sensitivity and accuracy of calling structural variations (SVs) can be affected. This motivated us to study the alignment of short reads that are associated not only with SV but also with single nucleotide variants (SNVs) and insert–delete (indel) variants.

Alignment tools were initially developed to align short reads allowing mismatches only. A number of such methods have been proposed, including SOAP (1), RMAP (2), Bowtie (3), PerM (4) and BatMis (5). Although they are generally fast, they will miss capturing the wide spectrum of non-SNVs that have been shown to represent 7–8% of human polymorphisms (6). As increasing evidences show that indels are involved in a wide range of diseases (7), mismatch aligners are unsuitable to be used in the studies of such biologically important events.

To align reads that span across indels, gapped aligners were proposed. Existing gapped alignment methods mostly use the seed-and-extend approach by first aligning a part of the read to obtain preliminary seeding locations for the queried read. Different gapped aligners use different seeding techniques, including contiguous exact match seeds (BLAT (8), MegaBLAST, SeqAlto (9), YAHA (10), BWA-MEM (11)), mismatch-seeds (RMAP (2), Stampy (12)), spaced-seeds (Eland (13), PatternHunter (14), MAQ (15), ZOOM (16)) and q-gram filters (RazerS (17), SHRiMP (18), MASAI (19)). Next, the seed locations are extended to the full length of the read, allowing for gaps, and these alignments are reported to the users.

Current gapped aligners generally offer reasonable efficiency and accuracy. However, they assume that the parts of each read used to obtain the preliminary seeding locations to have a small number of mismatches in them. This assumption will bias the alignments. Our analysis shows that the majority of the reads with incorrect alignments are (1) reads whose seeds have many mismatches or (2) reads rescued by incorrect pairing the paired reads. (1) is an unfavorable consequence of the seed-and-extend approach. When there are indels or too many mismatches in the read, the aligners will misalign the read due to incorrect seeding of candidate locations. (2) is due to biased pairing methodologies that over-rely on the estimated/given nominal insert sizes of the paired-end libraries. With existing approaches, paired reads that span over an SV (i.e. the aligned locations of the two reads are not within the expected insert-size) can be misaligned to other genomic locations where they can be concordantly aligned instead. This bias will affect alignment and adversely impact variant-calling performance.

Although misalignments of reads are low in general, it is important to resolve them as they are most-likely to be variant-spanning. As such, our variant of seed-and-extend gapped aligner, BatAlign, was developed to offer accurate alignments of reads spanning across SNVs, indels and SVs. Unlike existing seeding strategies, BatAlign allows high mismatches and a gap in the seeding regions of the read. It utilizes two strategies called ‘Reverse-alignment’ and ‘Deep-scan’ to find confident seed locations for reads. It also performs unbiased mapping of paired reads to avoid misaligning SV-spanning reads.

The organization of this paper is as follows. We first describe the simulation of data and how the performances of aligners are being compared with one another. Next, we describe the routines being implemented in BatAlign.

In the Discussion and Results section, we first touch on the inadequacies on current seed-and-extend methodologies, then we compare the performance of BatAlign with some published methods over a wide range of data sets: ART-simulated data sets, indel-aberrant data sets, simulated paired-end data sets, RSV-simulated SV-data sets and real data sets. Overall, the results show that BatAlign had the highest F-measure for aligning reads that contain variants or span across genomic breakpoints among the compared methods.

## MATERIALS AND METHODS

### Methods of experiments

#### Compared methods

We have used the following gapped alignment tools for comparison: BatAlign, Bowtie2 (2.0.6), BWA-Short, BWA-SW (0.6.1-r104), GEM (third release), SeqAlto (0.5-r123) and BWA-MEM (0.7.5a). These aligners are widely used and feature a wide range of mapping techniques. For each tool, the reference genome was indexed with default indexing parameters. hg19 reference genome was used for all experiments in this paper. All experiments were run on a Linux workstation equipped with Intel X5680 (3.33 GHz) processor and 16GB RAM.

#### Simulation of data

We generated four classes of simulated data. The first class mimicked Illumina-like reads, the second class has one indel in each of its reads, the third class is ‘paired’ reads and the last class is from an RSV-rearranged (20) genome. The first class of reads was generated by ART (21) from hg19 (excluding non-chromosomal sequences). We have chosen ART for our study since the substitution errors were simulated according to empirical, position-dependent distribution of base quality scores; it also simulates insertion and deletion errors directly from empirical distributions obtained from the training data from the 1000 genomes project (22). Empirical read quality score distributions were provided for read lengths 75, 100 and 250 bp (these are the longest read lengths made available by ART). We have capped the number of mismatches and indels (SNVs or base-call errors or gaps) in the simulated reads at 7%.

The second class of reads was used to demonstrate the performance of BatAlign on aligning reads with indels. The average density of an indel is 1 in ∼7.2 kb (23) so we simulated indels with ART at a much higher rate of 0.1% into two data sets (one each for insert- and delete-type of gap).

The third class of reads was used to demonstrate the efficacy of mate-pair information on the paired-end mapping mode of the compared programs. Six sets of 1 million reads were created. Each set consisted of 2 × 500 k x (75/100/250) bp x (concordant/discordant) reads. The first set consisted of concordant paired-end reads with a mean insert size of 500 bp and a standard deviation of 50 bp. The second set consisted of discordant paired-end reads, where the ‘left’ and ‘right’ ends of the paired reads were simulated from chromosome 1 and chromosome 2 of hg19, respectively. This class of reads was used to demonstrate the robustness of BatAlign when aligning reads with mate-pair information in the presence of genomic SVs.

The fourth class of reads was used to gauge the performance of aligners on SV-spanning reads. A total of 3760 SVs of insertions, deletions, duplications, inversions and translocations were simulated using the RSVsim package in the Bioconductor (24). Reads were simulated from the rearranged genome to a depth of 30X and aligned to the hg19 reference genome. The resulting alignments were subsequently applied with BreakDancer (25) to call out putative SVs and were validated against the oracle information from the simulator.

As simulated data come with oracle information, we have used the F-measure to gauge the performance of the methods: we define sensitivity (SEN) = TP/(TP+FN), accuracy (ACC) = TP/(TP+FP) where TP, FP and FN are true-positives, false-positives and false-negatives, respectively; F-measure = 2(SEN*ACC)/(SEN+ACC). As we do not have true-negatives in our simulated experiments, accuracy will be used interchangeably with specificity.

#### Comparison of alignment performance by stratifying against all reported mapQ scores

As the original locations of simulated reads were known, we have assessed the sensitivity and accuracy of each method using simulated reads in this section. For each method and each data set, we discarded mappings with mapQ = 0 for all methods as they were deemed ambiguous. Then, we recorded the cumulative number of correct and wrong alignments by their respective decreasing mapQ and plotted these results in a form similar to an ROC curve; the corresponding cumulative number of correct and wrong alignments at a particular mapQ cut-off will be the respective x-axis and y-axis values for a single data point on the Receiver Operating Characteristic (ROC) curve. In addition, due to the inability to align some indels to their exact locations and the presence of soft-clippings, an alignment will be considered as a correct mapping if the leftmost position was within 50 bp of the position simulated by the simulator on the same strand.

For real data sets, to address the lack of oracle information, we have mapped the paired-end reads as single-end reads and calculated the fraction of reads that were mapped concordantly. We consider a pair of reads to be concordant if they have the correct orientation and maps within 1000 bp of each other with a mapQ > 10. (The distance 1000 is chosen since Illumina GA II machines normally cannot sequence paired-end reads from DNA fragments of size longer than 1000 bp.) If both ends of the paired-end reads are mapped but are not located within a distance of 1000 bp to each other, they will be marked as discordant mappings. To plot the full spectrum of concordance/discordance in our experiments on real data set for the ROCs, we recorded the number of concordant and discordant alignments stratified by the mapQ score of the ‘head’ read. We must also emphasize that although the rate of concordant mappings was taken as a performance measure for aligning real reads, it can only give a lower bound of performance when used on mapping data sets of expectedly high paired-end concordance rates. Mapped reads with unmapped mate/pair-read will not be considered as they only form a minimal portion of the mappings and there are no oracle data to readily verify the correctness of their alignments.

#### Method of cross-comparison

It was noted that GEM is the only method among the six compared methods which does not calculate a mapQ for its alignments. We run the default modes of the compared programs unless otherwise stated. We have also adopted the performance measure ‘first correct’ (or best) alignments from GEM's paper into our experiments to make sure our comparisons were extensive and correct.

In this paper, we have compared the full spectrum of mappings by stratifying alignments by their reported mapping quality scores. However, it is hard to compare the absolute differences in performance between methods as the calculation of mapQ of an alignment differs from one method to another. To resolve this problem and to present the relative differences in the performances numerically between the different methods, we will have to pick a baseline performance indicator. For instance, we can compare the different rates of sensitivity of the methods at similar rates of specificity while using the program with the best specificity as a baseline performance indicator for sensitivity. In general, more false-positive mappings will come with increasing sensitivity. Hence, we picked and compared the sensitivity and specificity of the various methods as described to remove bias due to the calculation of mapQ.

### Our proposed solution: BatAlign = (Reverse-alignment + Deep-scan) + Unbiased mapping of paired reads

To align a read, existing approaches first find putative hits of short seeds from the query read. These putative partial hits are usually exact or 1-mismatch occurrences with respect to the reference genome. When there are high mismatches and/or indels in the read, it is likely that the seeded locations do not represent the original location of the queried read. To address the problem of missing hits from using low edit-distance short seeds, BatAlign uses high edit-distance in a long-seed (five mismatches, one gap and 75 bp) instead to search for a global base-call-quality-aware least-cost hit in the reference genome. To find the least-cost hits, BatAlign uses ‘Reverse-alignment’ to enumerate putative candidate hits in increasing order of alignment cost (i.e. increasing number of mismatches and gaps). Since the hit having a minimum number of mismatches may not be correct (as shown in the Discussion and Results section), ‘Deep-scan’ was developed to selectively scan deeper into the search space of putative hits even after the least-cost hit has been found. The alignments of all candidate hits reported by ‘Reverse-alignment’ and ‘Deep-scan’ will be extended to their original full read-length. Then, base-call-quality-aware scores for these hits are computed. For the single-end mode, BatAlign will report the hits in the order of this quality-aware score to the users. For the paired-end mode, BatAlign will align both reads in the paired-read independently as if they were from a single-end sequencing experiment. Next, BatAlign will report the alignments for the paired-reads that best represent the estimated insert-size of the prepared library.

### Details of algorithms in BatAlign

#### Problem definition and overview of the method

The problem of mapping genomic reads is defined ideally as follows: given a set of genomic reads, find the origin of each read in the reference genome, along with their correct alignments. However, in practice, this problem cannot always be solved and we have to resort to finding the most likely point of origin and alignment for each read.

The outline of BatAlign algorithm is as follows. As a pre-processing step, a one-time indexing of the reference genome is done. Next, it will start scanning for the most probable hits of the read in the reference by using ‘Reverse-alignment’. ‘Deep-scan’ is then applied to scan and pick the most probable hit of the read from the reference genome. BatAlign then calculates a mapQ score for this hit and reports it. Below, we will discuss the novel components that aid BatAlign to gain accuracy and efficiency.

### Reverse-alignment

Seed-based aligners search for candidate hits of its seeds; then, these hits are extended and the best alignment is selected based on a set of pre-defined criterion. In contrast, ‘Reverse-alignment’ does the opposite by searching for the best possible hits in the reference first.

With a set of match/mismatch/gap scores assigned, we pre-compute the combination and the order of matches/mismatches/gaps that each ‘step’ of the scanning routine will need to scan the reference genome with. Reverse-alignment scans the read in increasing ‘steps’ of alignment-cost. In this step, we pick non-overlapping 75-bp segments from the 5′ end of a read as seeds. For each hit of the seed, a maximum of five mismatches and one gap are allowed in a single-seeded region.

### Deep-scan

The best-scoring alignment need not be the correct alignment, even if it turns out to be the only hit with such a mismatches/gap combination. It is best if we can get the set of next-best alignments too. With these additional hits and using the quality information of the sequencing base-calls, we can better differentiate the correct hit from a pool of putative candidate hits. Furthermore, these extra hits will help BatAlign to assess the quality of the final alignment better as the mapping quality of the final alignment is computed from the two-best hits. If the first hits found during ‘Reverse-alignment’ are multiple hits, then we return all of these hits. Otherwise, if it is a unique hit pertaining to such a mismatch/gap combination, then ‘Deep-scan’ will be activated to scan for the next-best alignments.

### Handling long reads

For reads longer than or equal to 150 bp, we will split the read into non-overlapping 75-bp reads. Each of the 75-bp segments will be aligned as described above. For instance, for 250-bp reads, BatAlign will obtain three consecutive segments of a read starting from the first base of the read and map each of them individually. If the first or best hits from each segment are non-repetitive and fall within the locality of each other, we will try to align the original read onto this region of the reference. By doing this, we avoid realigning the original read to more than one location of the reference. However, if the first or best hits from each segment are repetitive or not mapped to the locality to one another, BatAlign will examine and align the whole read onto each of the putative locations reported by each of the segments. Among these alignments, the best-scoring hit is reported.

### Mapping with mate-pair information

BatAlign will first align all paired-reads in unpaired-fashion. If the top hits from each of the paired reads are confidently mapped and are within expected distance to each other, BatAlign will report this pair of alignments. However, if the reads cannot be paired up within the expected distance or one of the pair-reads is unmapped, SW-algorithm will be applied to the neighboring region of the anchored alignments to rescue the mate of the anchored reads. From here, we can calculate the mapQ for all the hits of the paired-reads. Instead of using just a cutoff for the alignment score of the rescued read, we also try to discriminate the goodness of the rescued seeds using mapQ, alignment score and mate-pair information simultaneously. Thus, for unbiased detection of SVs caused by discordant paired-reads, the calculated alignment scores will precede mate-pair information.

### Supplementary alignment of SV breakpoint-spanning read

The part of the read, which spans across a genomic rearrangement breakpoint, will have many mismatches with respect to the reference genome, possibly incurring a negative alignment score, and will be soft-clipped away. For example, a CIGAR alignment string of ‘65M35S’ is possible for a length-100-bp read. In this example, we might be clipping away useful information, which can be crucial to identifying the partnering breakpoint of an SV.

Hence, BatAlign will realign the clipped portion from the primary alignment of a read whenever the clipped length of the alignment exceeds 20 bp. A fast 0-mismatch scan is applied to the last 20 bp of the clipped bases to find the candidate locations near potential SVs. Next, the same read will be realigned locally to the candidate locations to recover the auxiliary alignments. The chosen auxiliary alignment should complement the primary alignment of a read and together with the primary alignment, be able to represent the full length of the original read. In other words, the primary and auxiliary alignment can be used interchangeably for the same read.

### Faster semi-global alignment and SW alignment

After the seed alignments are found for a read, BatAlign can perform either SW alignment or semi-global alignment to extend the alignment of the read. We have devised a semi-global alignment method that is faster than SW-alignment by ∼30%, and the default mode of BatAlign is to extend the seeds using this semi-global alignment method. When the alignment score of the semi-global alignment drops below 90% of the maximum alignment score (i.e. the score for an exact match), an SW-alignment is done. If the user wants to perform SW-extensions only, an option is provided to do so. Below, we describe the SW alignment and the semi-global alignment methods.

The SW alignment is SIMD accelerated via SSE2 instructions. Our implementation is based on an extension of SSW library (26) that modifies Farrar's method (27). This algorithm determines the best alignment in two steps: first it will calculate the best SW-score and then it will perform a banded SW alignment to get the optimal trace-back of the alignment from the Dynamic Programming (DP) table.

**Figure 1.**
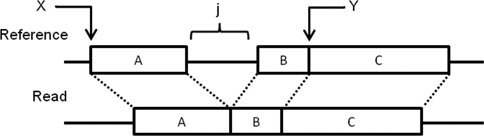
Example of recovering a delete in a reference from a read.

For the semi-global alignment, we designed a new algorithm assuming that there is at most one gap in the read. The algorithm will divide the read into two halves and first assume that the indel is in the left half. If the indel is in the left half, the right half of the read must align to the reference with only mismatches. Figure 1 shows the situation for the case of a deletion. The right half of the read (part C) maps to location Y in the genome. Part A of the read maps to location X in the reference. Location X will be found by the BatAlign algorithm where a seed of length |*R*|/2 will be mapped. Assume we allow a maximum of *d* bp for the indel, we will set *j* = 1…*d*, and map part C of the read *j* bp away from part A of the read. See Supplementary Sections 1 and 2 for the details on how BatAlign handles indels in different parts of the read and for the analysis of the novel data structure behind this routine, respectively.

### Accelerating alignment

The speed of the algorithm is improved by limiting the number of SW alignments performed for each read. Another way is to stop performing SW extensions when the best alignment score has failed to increase after a determined number of attempts. To trace back the optimal alignment path in the DP table, we need to perform a non-SIMD-banded version of SW algorithm. This step is time consuming. However, we can skip this step if the SW score of the alignment falls below the current best SW score.

### Alignment score and mapping quality

Sequencing data can contain a per-base quality score that indicates the reliability of a base call. If the probability of a base call at position *i* being correct is *P*[*i*], the quality score *Q*[*i*] assigned to location *i* is given by the equation *P*[*i*] = 1 − 10^−*Q*[*i*]/10^. Assuming that there is no bias to a particular set of nucleotides, the probability of a base being miscalled at location *i* can be calculated by the formula 1 − *P*[*i*]/3. For a given alignment, we compute an alignment score based on an affine-gap scoring scheme, where the score for a match or a mismatch at *R*[*i*] is the Phred scaled value of *P*[*i*]. See Supplementary Section 2 for details on how the mapQ is calculated from alignment scores.

## DISCUSSION AND RESULTS

Mapping biases, which occur in genomic regions with strong homology to other genomic locations (28), contribute to erroneous callings of SNVs, indels and SVs. This problem should be given more attention as misalignments by a particular aligner tend to be recurrent among reads that share similar genomic contexts. As such, we are strongly motivated to study the alignment performance of officially published methods on reads with and without spanning variants. Based on our study, we developed BatAlign for accurate, sensitive and efficient alignment of next generation sequencing (NGS) reads.

### Simulation study showing that existing methods have difficulties mapping reads with high mismatches or located near SVs

The alignment of reads in the presence of SNVs, and/or SVs, still remains challenging despite developments already made by published aligners. This section intends to study the alignment accuracy of existing published methods using simulated reads that span across genomic regions with a high number of SNVs or near SVs.

#### Simulated reads with k mismatches can be mapped with less than k mismatches

Mismatches (like Single Nucleotide Polymorphisms (SNPs)) can cause misalignments of reads to homologous genomic regions, especially when reads are sequenced from highly polymorphic regions. We simulated reads (see ‘Simulation of data’) to study the effects of mismatches in producing misalignments. For each read, we reported the lowest-mismatch unique hits (using BatMis (5), an exact k-mismatch alignment algorithm). We then compared the number of mismatches at which the reads were simulated with (we call this value *A*) and mapped at (we call this value *B*). Interestingly, if *A* = *B*, the respective alignments from BatMis were mapped correctly. However, when *A*≠*B*, the mappings were wrong, as it must be so due to being aligned to a location different from where it was simulated.

We should note that with the increase of simulated mismatches in a read, the occurrences of it being misaligned with a lesser number of mismatches also increase; statistically, this is true as mismatches act as wild cards in string-matching problems. From the mappings of BatMis, the rates of misalignment for reads simulated with one to five mismatches increased from 0.3 to 0.9%, respectively. This result implies that, in SNV-aberrant genomic reads, it is unwise to always pick the lowest-mismatch hit as it might misrepresent the original location of a read.

**Figure 2.**
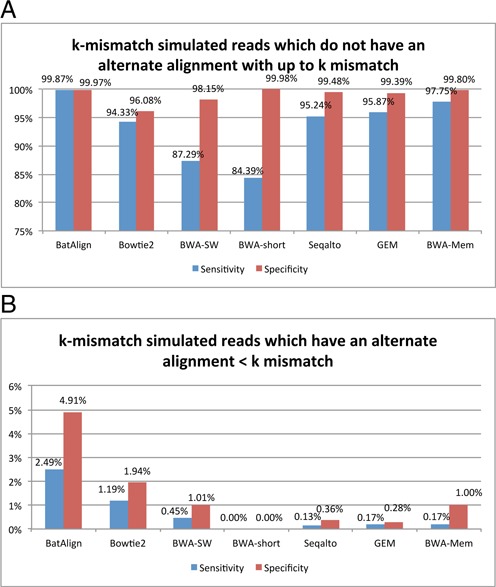
Panel (**A**) The sensitivity and specificity of compared methods on k-mismatch reads which can be mapped uniquely with k-mismatch. Panel (**B**) shows similar statistics to Panel (A) by mapping k-mismatch reads which have alternate unique alignment of ≤k-mismatch.

To further investigate the impact of high-mismatch reads on the performance of the current published methods, we procured two groups of reads from the current set of simulated reads. The first and second groups consisted of k-mismatch reads that can be mapped uniquely by k-mismatch and less than k-mismatch, respectively. On the first group of reads, all the compared published methods have an average sensitivity of ∼90% and the specificity approaches 100%. However, on the second group of reads, both sensitivity and specificity never exceeded 2% (see Figure 2). This highlights the difficulty faced by current methods on mapping high-mismatch reads and will later be resolved by using BatAlign's ‘Deep-Scan’.

#### Mate-pair information can falsely disambiguate alignments

Mate-pair information is useful in aligning two individually repetitive mate-paired reads unambiguously to the locality of each other in the reference genome. An ideal aligner should be able to align concordant and discordant paired-reads without bias, i.e. same rates of specificity while maintaining high sensitivity on mapping these two types of reads. (A concordant paired-read is a pair of reads that are sequenced from the vicinity of each other, within the expected wet-lab insert-size, with respect to the reference genome; otherwise, it is a discordant paired-read.) However, if mate-pair information is used too aggressively, an aligner might wrongly align a pair of discordant read-pair concordantly onto the reference genome.

**Figure 3.**
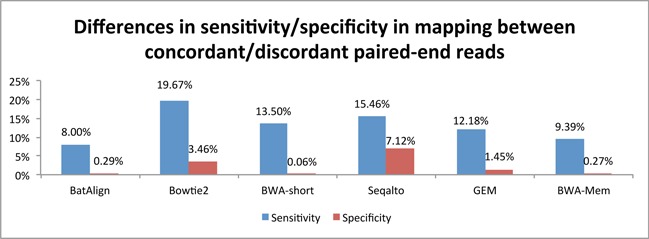
The differences in sensitivity and specificity between mapping paired-end data sets with simulated concordant and discordant paired-end information.

We have studied the impact of mate-pair information on alignment performance by aligning two types of simulated paired-reads (see ‘Simulation of data’). The first set consists of paired-reads that were simulated with a mean insert-size of 500 bp (SD of 50 bp) and the other set consists of paired-reads simulated with each end of the paired-reads from different chromosomes. Figure 3 reports on the differences in mapping sensitivity and specificity of each published method between these two sets of reads. An ideal aligner should exhibit minimal performance shift between these two types of reads. However, we observed that the alignment performance of the compared methods varied greatly from one another between these two types of paired-reads. The estimated bias, between mapping concordant and discordant read-pairs, in terms of sensitivity and specificity ranged from ∼9.4 to ∼20% and ∼0.1 to ∼7.1%, respectively, among the compared methods.

### Evaluation on ART-simulated reads

**Figure 4.**
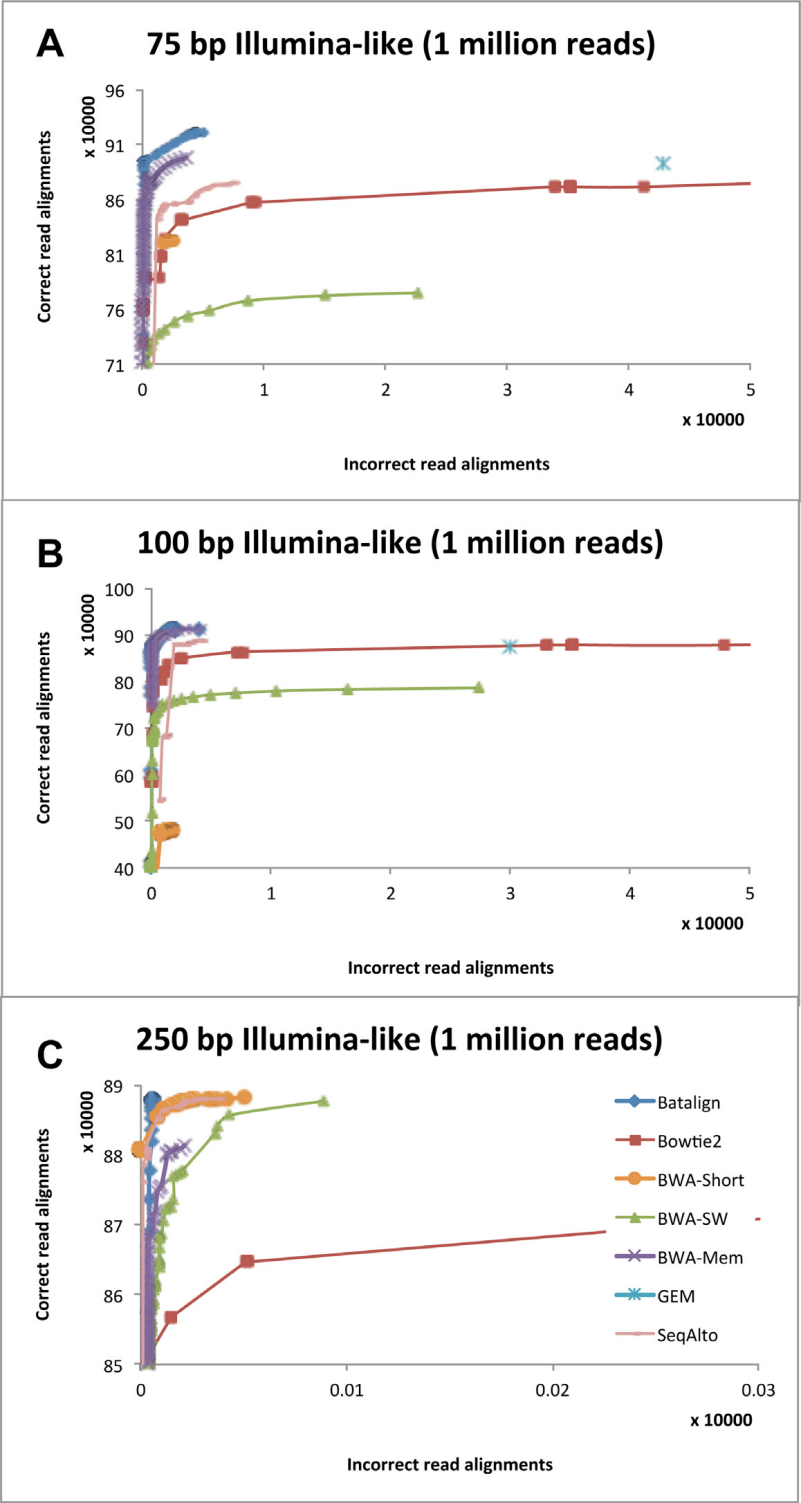
Sensitivity and accuracy for aligning simulated reads from ART. Cumulative counts of correct and wrong alignments from high to low mapping quality for simulated Illumina-like (**A**) 75-bp, (**B**) 100-bp and (**C**) 250-bp data sets.

To evaluate the performance of BatAlign on aligning reads, we compared it with six other officially published methods. We used ART (21) to simulate three data sets of 75, 100 and 250-bp read-lengths. Then, the reads in these data sets were aligned using the different methods. Figure 4 depicts the ROC plots on the ART-simulated data sets. Validation on the alignments showed that BatAlign has a better performance than the other compared methods in terms of both sensitivity and specificity over a large range of mapQ on the 75/100/250-bp data sets. We also cross-compared the methods as described in ‘Compared methods and method of cross-comparison’ and presented their respective sensitivity and accuracy in Table 1. As shown in Table 1, BatAlign consistently outperformed the other compared methods in terms of sensitivity and specificity on simulated reads of various read-lengths.

**Table 1.** Cross-comparison of sensitivity at similar specificity and vice versa for simulated data sets of 75/100/250 bp

Simulated data	Bat-Align		Bowtie2		BWA-SW		SeqAlto		BWA-short		BWA-MEM	
	SEN	ACC	SEN	ACC	SEN	ACC	SEN	ACC	SEN	ACC	SEN	ACC
75 bp	**91.0**	**99.998**	84.1	99.987	74.9	97.168	85.5	99.862	82.2	99.944	89.5	**99.998**
100 bp	**91.1**	**100.0**	83.7	99.992	75.8	96.644	87.7	99.786	47.8	-	90.2	99.999
250 bp	**88.8**	99.999	85.1	99.891	86.1	99.996	88.2	99.999	88.5	**100.0**	87.1	99.998

SEN is sensitivity.

ACC is specificity.

Best performance pertaining to each read-length are in BOLD.

Similar to the experiments performed in GEM's paper, we also validated the top 10 hits reported by each method. The complete breakdown of this validation by the first (or best) alignment, as ordered by their respective aligner, can be found in Table 2. From Table 2, we can see that BatAlign has reported the most number of correct hits as top-ranked hits in our simulated data. See Supplementary Table S3.1 for the validation results of the top 10 hits on 75- and 250-bp data sets.

**Table 2.** Number of first (or best) alignment reported by various methods on simulated 100-bp data set

Rank	100-bp data set #Correct hits										
Aligner	1	2	3	4	5	6	7	8	9	10	Sum of correct hits
BatAlign	924272	7599	941	728	851	332	182	108	101	63	935177
Bowtie2	866310	8685	2833	948	254	110	69	42	23	5	879279
BWA-SW	794661	7	0	0	0	0	0	0	0	0	794668
Seqalto	890336	1821	515	194	91	44	38	11	3	8	893061
GEM	875333	10327	3533	1445	638	377	283	193	163	178	892470
BWA-Short	484558	5207	1747	662	211	112	79	48	20	13	492657
BWA-MEM	912562	0	0	0	0	0	0	0	0	0	912562

### Evaluation on simulated indel-aberrant reads

The reads generated by ART have less than 0.01% probability of containing an indel. Therefore, ART-simulated data sets only show the performance of the methods on reads containing mismatches and SNVs. We used ART to spike in either inserts or deletions into each data set at a rate of 0.1% to further gauge the performance of BatAlign on indel-aberrant data.

Since BatAlign allowed one gap in the seed region, BatAlign can seed locations for an indel-read with high accuracy and without bias for mismatch-stricken locations which will cause indel-reads to be misaligned. On this read class, BatAlign achieved the highest F-measure of 92.0 and 91.9% on the ‘delete’ and ‘insert’, respectively. BWA-MEM also performed well on this read-class with an F-measure of 90.8% due to the stitching of multiple maximal exact matching read-segments into the final alignment of a read. See Supplementary Table S3.2 for the detailed results of alignments on the indel-aberrant data sets.

The results from aligning on ART-simulated and indel-aberrant data sets showed that BatAlign has better performance than the other methods on aligning a general ART-simulated data set of reads containing a mixture of mismatches and indels. Thus, BatAlign can be used to identify a broad spectrum of short-range intra-chromosomal variants, in the presence of sequencing errors. We will discuss the performance of all compared methods in identifying long-range intra/inter-chromosomal variants in the next section.

### Evaluation on concordant- and discordant-paired reads

Another issue of existing methods is that they may over-aggressively assume paired-reads to be concordant on the reference genome. In this section, we present the results on mapping concordant (emulating a normal genome) and discordant (emulating large deletions and SVs in a diseased genome) simulated reads using the paired-end mapping mode available in the compared methods.

On the concordant paired-end data set, BatAlign, Bowtie2, BWA-SW, GEM, BWA-Short, SeqAlto and BWA-MEM reported sensitivities with their corresponding specificities of 98.4% (99.891%), 91.1% (92.601%), 93.0% (98.711%), 97.4% (98.183%), 60.2% (99.702%), 96.2% (99.881%) and 98.2% (99.834%), respectively. When mapping concordant paired-reads, almost all the compared methods have similar accuracy. In contrast, with the discordant paired-end data set, the sensitivity dropped for all the compared programs. Despite the drop in alignment performance, BatAlign still reported the highest sensitivity and specificity. On the discordant paired-end data set, at mapQ > 0, the sensitivity of the compared methods with their corresponding specificity for BatAlign, Bowtie2, BWA-SW, GEM, BWA-Short, SeqAlto and BWA-MEM is 90.4% (99.571%), 71.5% (96.058%), -(-), 85.2% (96.735%), 46.7% (99.646%), 80.7% (92.759%) and 88.8% (99.565%), respectively. In general, BatAlign has the highest F-measure of 99.1 and 94.8% for the concordant and discordant paired-end data sets.

**Figure 5.**
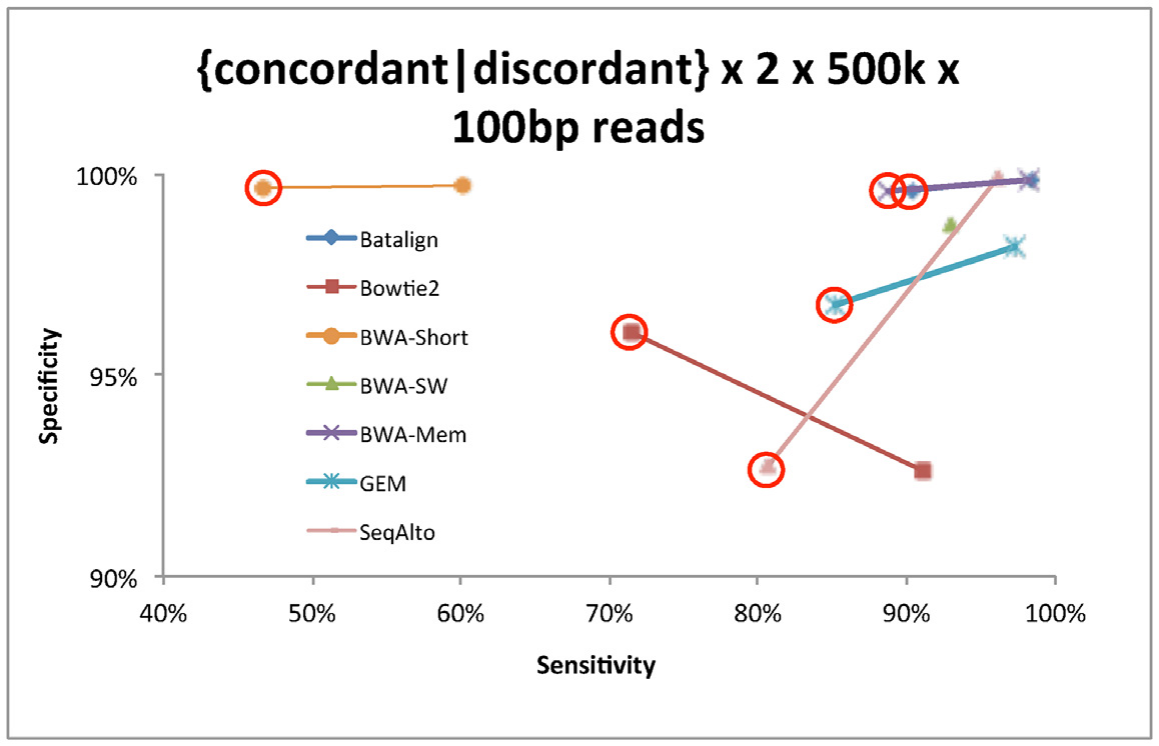
Sensitivity and specificity on mapping of concordant and discordant data sets using paired-end mapping mode of various methods. Data points circled in red depicts mapping performance on discordant data set. BWA-SW was unable to complete the alignment of 2 × 500 k x 100 bp discordantly paired reads and is plotted as a single data point.

The mapping performance on these two data sets from the compared methods is shown in Figure 5. The alignment performances on each of the two data sets, from the same method, were joined together by a line. One can infer the robustness of paired-end mapping mode of a method from the interpolation of the line that joins the paired data points of the corresponding method in Figure 5. Thus, Figure 5 graphically shows how biased a method can be when aligning with mate-pair information. Overall, BatAlign was observed to have the smallest fluctuations in its F-measure, by only ∼0.6%, between the two data sets.

From Figure 5, an interesting trend of results was observed for the compared programs excluding BatAlign. The initial observation was that methods which had lower specificity on the concordant-paired data set will generally suffer a smaller drop in specificity on the discordant-paired data set. For instance, Bowtie2 used to have a specificity of 92.601% on the concordant set but the specificity improved to 96.058% on the discordant set. The inverse of the initial observation on the results was also true. SeqAlto used to have the highest specificity of 99.881% on the concordant set but its specificity suffered the largest drop of 7.122–92.759% on the discordant paired-end data set. These fluctuations in specificity are due to the aggressiveness of the pairing algorithms in the various methods to map paired-end reads close to each other on the reference genome.

The results in this subsection were obtained from running data sets of 100 bp long. Experiments were also done using 75- and 250-bp data sets and the trend of results was consistent among all three data sets.

### Evaluation on reads from an RSVsim rearranged genome

Recent developments in SV callers have revolved around the usage of soft-clipped reads (29,30) and spanning read-pairs (25). By extracting soft-clipped aligned reads and spanning read-pairs (read-pairs that are aligned outside of the expected range of insert-size or/and with different orientations from what are designed in the wet-lab sequencing protocols) alignments, an SV can be inferred from such signatures using an SV-calling algorithm.

Due to efficiency reasons and insufficient soft-clipped alignments (due to the local-realignment strategy and alignment scores used) from some of the compared aligners, we have decided to use BreakDancer to call our putative SVs back from the respective sets of alignments to gauge their performance on recovering the SVs. In addition, Table 3 shows the robustness of the alignments if the samples were to be downsized to rates of 50 and 25% from the original simulated coverage depth of 30X. For the various downsampled data sets, the SVs called out from BatAlign's alignments have higher F-measures consistently. Apart from calling SVs, BatAlign also achieved similar trends of results from callings SNVs and indels. Results of these experiments are included in Supplementary Section 3.3.

**Table 3.** F-measures of SV-callings against oracle information from Bioconductor's RSVsim package at various downsampled rates of the data set from an original depth of 30X

Method	F-measures of SV-calling (at various downsampling rates)		
	25%	50%	100%
BatAlign	**79.87%**	**83.73%**	**89.08%**
Bowtie2	75.99%	78.56%	76.21%
BWA	3.02%	7.87%	15.84%
BWA-SW	73.39%	78.88%	83.68%
GEM	75.17%	80.08%	79.09%
SeqAlto	76.11%	83.34%	88.63%
BWA-MEM	72.68%	70.87%	80.94%

Best performance pertaining to each downsampled rate are in BOLD.

### Evaluation on real reads

**Figure 6.**
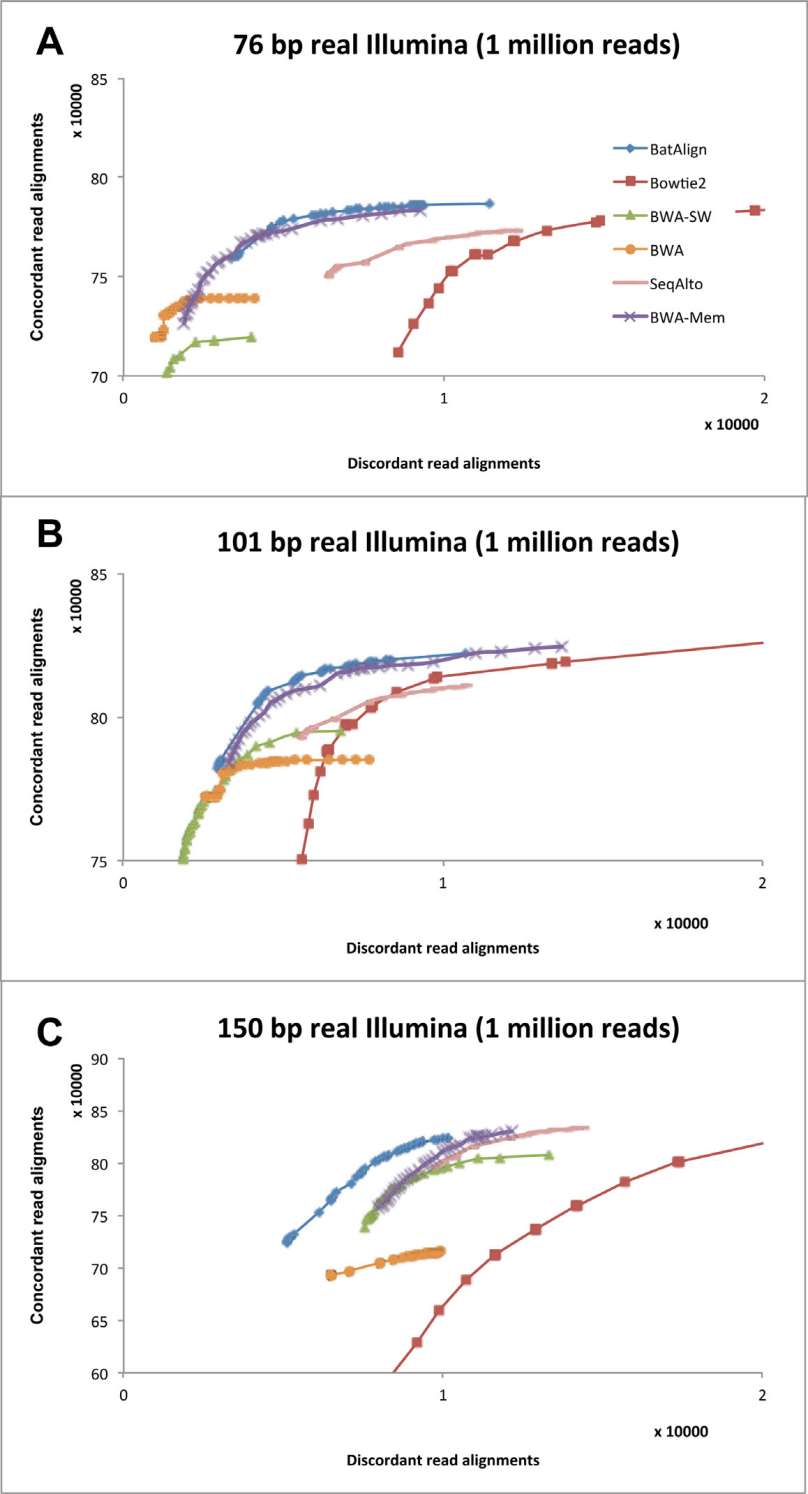
Concordance and discordance rates of alignments on real reads. Cumulative counts of concordant and discordant alignments from high to low mapping quality for real sequencing reads (**A**) 76 bp, (**B**) 101 bp and (**C**) 150-bp data sets.

We have downloaded 2 × 76 bp (SRA accession DRR000614, Sample: NA18943), 2 × 101 bp (SRA accession SRR315803, Sample: NGCII082 Mononuclear blood) and 2 × 150 bp (SRA accession ERR057562, Sample: ERS054071) paired-end data sets. The sequencing platform used for the downloaded data sets was Illumina Genome Analyzer IIx for the 76/101-bp data set and Illumina MiSeq for the 150-bp data set. We evaluated alignment performance on real data by performing single-end read mapping on paired-end data sets. Subsequently, we used the concordance and discordance mapping rates from the alignments to estimate the correct and wrong alignment rates (see ‘Comparison of alignment performance using ROC graphs’ for more details on the definition of concordance and discordance). In order to minimize error in estimating alignment performance by using concordance information from the alignments, we have only used sequencing data from non-cancerous origins. Figure 6 depicts the ROC plots on the real data sets. Similar to our results on simulated data, BatAlign has reported more concordant and less discordant alignments on the tested real data sets over a large range of mapQ scores.

To verify if better mapping can improve variant calling, we apply different variant callers to the alignments of reads from some real data set. First, we inspected the discordant read-pairs spanning validated SVs across various downsampled rates of a real data set (Accession: ERP001196, Patient Sample: 46T, Read Format: 2 × 90 bp, Nominal Insert-size: 170 bp). This patient sample was chosen as it contained a higher number of polymerase chain reaction (PCR)-validated genomic rearrangements compared to the other samples in (31). In Table 4A, we reported the number of SVs being supported by the alignments from the respective aligners. Across different downsampled rates of the data set, BatAlign was able to recall the most number of PCR-validated SVs across all of them. Table 4B reports on the number of candidate SVs being called out by BreakDancer, when compared with the number of validated SVs, we can estimate specificity of the alignments which spanned outside of the expected sequenced insert-size. As compared to the second-best method, BatAlign produced 73.3% less callings but was still able to recall 4.3% more PCR-validated SVs. From this, we can infer that BatAlign is both sensitive and specific on aligning SV-spanning reads.

**Table 4A.** Comparison on the number of SVs recalled across various sub-sampled data of published and validated SVs of Patient 46T through manual counting of supporting real pairs

Methods	Intersect with published 46T data (at various ‘x’ downsampling rates) - PE170-insert_size					
	1x	2x	3x	4x	5x	6x
BatAlign	121	114	100	83	68	59
Bowtie2	98	71	64	62	57	55
BWA	78	64	62	59	57	54
BWA-SW	116	104	81	71	65	58
GEM	80	66	64	63	60	55
SeqAlto	106	77	65	62	59	56
BWA-MEM	116	103	82	68	66	58

**Table 4B.** Total number of putative SVs called from across various sub-sampled data of Patient 46T

Methods	Number of SVs called by Breakdancer across downsampled rates					
	1x (base)	2x	3x	4x	5x	6x
BatAlign	39376	13105	7758	5180	3703	2800
Bowtie2	103721	29114	20133	15272	12332	10399
BWA	45184	9016	5129	3284	2330	1739
BWA-SW	52380	34729	22040	14931	10853	8259
GEM	16768	6449	3271	1969	1315	962
SeqAlto	56801	16760	10147	6831	4939	3829
BWA-MEM	68227	15062	9184	6135	4344	3237

The library used had ∼20X in sequencing depth. A total of 126 validated SVs (31) were used for this comparison.

Apart from calling SVs on real data, BatAlign also achieved similar results in the aspect of callings SNVs, using SAMtools (32) and VCFtools (33), on Patient 11T (sample was selected based on similar reasons as before for Patient 46T). Detailed results of these experiments were included into Supplementary Section 4.2.

### Evaluation on running times

Up till now, we reported on the F-measures (simulated data), concordance/discordance and variant-calling performance (real data) of the compared methods. BatAlign was generally observed to have the highest performance on these mentioned measures among the compared methods. BatAlign was developed to focus primarily on reporting accurate alignments and is also reasonably efficient. Table 5 shows the relative runtimes and speed factors among the programs. (See Supplementary Section 5 for the usage of parameters for all the compared methods.) The default setting of BatAlign ran faster than most of the existing published aligners except BWA-MEM, GEM and Bowtie2. As some users need fast alignment, we have provided additional single-end modes (Fast and Turbo) by reducing the search space of BatAlign. The additional modes were more accurate than existing aligners (see Supplementary Section 6) while the running times were made to be comparable with GEM and Bowtie2.

**Table 5.** Comparison of running times across all compared programs on 1 million reads from SRR315803

Program	Runtime (s)	Speedup factor
BatAlign - Default	583	1.2
BatAlign - Faster	481	1.4
BatAlign - Fastest	331	2.0
Bowtie2	459	1.5
BWA-Short	598	1.1
BWA-SW	639	1.1
**GEM**	**214**	**3.2**
SeqAlto	677	1.0
BWA-MEM	219	3.1

Fastest speed up in bold.

## CONCLUSION

We presented a method BatAlign, for the gapped alignment of short reads onto a reference genome with improved accuracy and sensitivity. The mapping strategies discussed in the Materials and Methods section, such as ‘Reverse-alignment’, ‘Deep-scan’ and ‘Mapping with mate-pair information’, produced mappings with increased accuracy when compared with other methods in simulated data (ART-simulated, indel-aberrant, paired-end, variant-spanning). In addition, BatAlign also aligned over sites of PCR-validated SVs and SNVs on real data more robustly over various downsampling rates of the input data. A new ‘faster semi-global alignment algorithm’ and other heuristics have also been used to replace the traditional SW routine to speed up BatAlign. In general, BatAlign is an improved aligner for accurate gapped alignment of DNA sequencing reads.

Recently, a number of aligners such as YAHA (10) and CUSHAW2 (34) were developed to handle long reads (500 bp or more). A possible future work is to develop an accurate tool for the alignment of long reads.

## SUPPLEMENTARY DATA

Supplementary Data are available at NAR Online.

SUPPLEMENTARY DATA

## References

[1] Li R., Li Y., Kristiansen K., Wang J. (2008). SOAP: short oligonucleotide alignment program. Bioinformatics.

[2] Smith A.D., Xuan Z.Y., Zhang M.Q. (2008). Using quality scores and longer reads improves accuracy of Solexa read mapping. BMC Bioinformatics.

[3] Langmead B., Trapnell C., Pop M., Salzberg S.L. (2009). Ultrafast and memory-efficient alignment of short DNA sequences to the human genome. Genome Biol..

[4] Chen Y., Souaiaia T., Chen T. (2009). PerM: efficient mapping of short sequencing reads with periodic full sensitive spaced seeds. Bioinformatics.

[5] Tennakoon C., Purbojati R.W., Sung W.K. (2012). BatMis: a fast algorithm for k-mismatch mapping. Bioinformatics.

[6] Bhangale T.R., Rieder M.J., Livingston R.J., Nickerson D.A. (2005). Comprehensive identification and characterization of diallelic insertion-deletion polymorphisms in 330 human candidate genes. Hum. Mol. Genet..

[7] Yang H., Zhong Y., Peng C., Chen J.Q., Tian D. (2010). Important role of indels in somatic mutations of human cancer genes. BMC Med. Genet..

[8] Kent W.J. (2002). BLAT—the BLAST-like alignment tool. Genome Res..

[9] Mu J.C., Jiang H., Kiani A., Mohiyuddin M., Bani Asadi N., Wong W.H. (2012). Fast and accurate read alignment for resequencing. Bioinformatics.

[10] Faust G.G., Hall I.M. (2012). YAHA: fast and flexible long-read alignment with optimal breakpoint detection. Bioinformatics.

[11] Li H. (2013). Aligning sequence reads, clone sequences and assembly contigs with BWA-MEM.

[12] Lunter G., Goodson M. (2011). Stampy: a statistical algorithm for sensitive and fast mapping of Illumina sequence reads. Genome Res..

[13] Cox A. (2006). ELAND: Efficient Local Alignment of Nucleotide Data.

[14] Ma B., Tromp J., Li M. (2002). PatternHunter: faster and more sensitive homology search. Bioinformatics.

[15] Li H., Ruan J., Durbin R. (2008). Mapping short DNA sequencing reads and calling variants using mapping quality scores. Genome Res..

[16] Lin H., Zhang Z.F., Zhang M.Q., Ma B., Li M. (2008). ZOOM! Zillions of oligos mapped. Bioinformatics.

[17] Weese D., Emde A.K., Rausch T., Doring A., Reinert K. (2009). RazerS–fast read mapping with sensitivity control. Genome Res..

[18] Rumble S.M., Lacroute P., Dalca A.V., Fiume M., Sidow A., Brudno M. (2009). SHRiMP: accurate mapping of short color-space reads. PLoS Comput. Biol..

[19] Siragusa E., Weese D., Reinert K. (2013). Fast and accurate read mapping with approximate seeds and multiple backtracking. Nucleic Acids Res..

[20] Bartenhagen C., Dugas M. (2013). RSVSim: an R/Bioconductor package for the simulation of structural variations. Bioinformatics.

[21] Huang W., Li L., Myers J.R., Marth G.T. (2012). ART: a next-generation sequencing read simulator. Bioinformatics.

[22] Durbin R.M., Altshuler D., Abecasis G.R., Bentley D.R., Chakravarti A., Clark A.G., Collins F.S. (2010). A map of human genome variation from population-scale sequencing. Nature.

[23] Mills R.E., Luttig C.T., Larkins C.E., Beauchamp A., Tsui C., Pittard W.S., Devine S.E. (2006). An initial map of insertion and deletion (INDEL) variation in the human genome. Genome Res..

[24] Gentleman R.C., Carey V.J., Bates D.M., Bolstad B., Dettling M., Dudoit S., Ellis B., Gautier L., Ge Y., Gentry J. (2004). Bioconductor: open software development for computational biology and bioinformatics. Genome Biol..

[25] Chen K., Wallis J.W., McLellan M.D., Larson D.E., Kalicki J.M., Pohl C.S., McGrath S.D., Wendl M.C., Zhang Q., Locke D.P. (2009). BreakDancer: an algorithm for high-resolution mapping of genomic structural variation. Nat. Methods.

[26] Zhao M., Lee W.P., Marth G.T. (2013). SSW Library: an SIMD Smith-Waterman C/C++ library for use in genomic applications. PLoS ONE.

[27] Farrar M. (2007). Striped Smith-Waterman speeds database searches six times over other SIMD implementations. Bioinformatics.

[28] Degner J.F., Marioni J.C., Pai A.A., Pickrell J.K., Nkadori E., Gilad Y., Pritchard J.K. (2009). Effect of read-mapping biases on detecting allele-specific expression from RNA-sequencing data. Bioinformatics.

[29] Wang J., Mullighan C.G., Easton J., Roberts S., Heatley S.L., Ma J., Rusch M.C., Chen K., Harris C.C., Ding L. (2011). CREST maps somatic structural variation in cancer genomes with base-pair resolution. Nat. Methods.

[30] Zhang Z.D.D., Du J., Lam H., Abyzov A., Urban A.E., Snyder M., Gerstein M. (2011). Identification of genomic indels and structural variations using split reads. BMC Genomics.

[31] Fernandez-Banet J., Lee N.P., Chan K.T., Gao H., Liu X., Sung W.K., Tan W., Fan S.T., Poon R.T., Li S. (2014). Decoding complex patterns of genomic rearrangement in hepatocellular carcinoma. Genomics.

[32] Li H., Handsaker B., Wysoker A., Fennell T., Ruan J., Homer N., Marth G., Abecasis G., Durbin R. (2009). The Sequence Alignment/Map format and SAMtools. Bioinformatics.

[33] Danecek P., Auton A., Abecasis G., Albers C.A., Banks E., DePristo M.A., Handsaker R.E., Lunter G., Marth G.T., Sherry S.T. (2011). The variant call format and VCFtools. Bioinformatics.

[34] Liu Y., Schmidt B. (2012). Long read alignment based on maximal exact match seeds. Bioinformatics.

