# Supplementary material for: BatAlign: an incremental method for accurate alignment of sequencing reads
Source: Nucleic Acids Res. 2015 Jul 13;43(16):e107. doi: 10.1093/nar/gkv533 (PMC4652746; doi:10.1093/nar/gkv533)
Supplement: SUPPLEMENTARY DATA [file supp_43_16_e107__index.html]

BatAlign: an incremental method for accurate alignment of sequencing reads — BatAlign: an incremental method for accurate alignment of sequencing reads — SUPPLEMENTARY DATA 

# BatAlign: an incremental method for accurate alignment of sequencing reads

## SUPPLEMENTARY DATA

- SUPPLEMENTARY DATA
