# Supplementary material for: BatAlign: an incremental method for accurate alignment of sequencing reads
Source: Nucleic Acids Res. 2015 Jul 13;43(16):e107. doi: 10.1093/nar/gkv533 (PMC4652746; doi:10.1093/nar/gkv533)

# Supplementary Materials

## 1 Preliminaries

We first briefly review some indexing data-structures: suffix array (Section 1.1) and Burrows-Wheeler Transform (Section 1.2). Then, a modified index is described in Section 1.3 that enables us to efficiently extract exact match hits of some fixed length strings.

### 1.1 The Suffix Array

Let $T[1..n]$ be a genome of length $n$ where the nucleotides are represented by characters taken from the alphabet $\Sigma = \{a, c, g, t\}$. We assume that a special character \$ appears at the end of $T$, and it is assumed to be lexicographically smaller than all the characters in $\Sigma$. The suffix array $SA_T[1..n]$ of $T$ is obtained by sorting the suffixes of $T$ in lexicographically increasing order, and setting $SA_T[i]$ to be the starting position of the suffix in the $i^{th}$ position in this sorted list. We will call each entry in $SA_T$ as an $SA_T$-value.

Let $P$ be a string. Suppose $SA_T[i]$ and $SA_T[j]$ are the lexicographically smallest and largest suffixes respectively, having $P$ as a prefix. We define the interval $[i, j]$ as the $SA_T$ range of $P$. The length of the $SA_T$ range of $P$ is $j - i + 1$. The entries $SA[i + k]$ for $k = 0, .., j - i$ gives all the locations in $T$ where $P$ occurs. Note that if the $SA_T$-range of $P$ happens to be an interval of the form $[i, i]$, $P$ occurs at a unique location in $T$.

### 1.2 The Burrows-Wheeler Transform

The Burrows-Wheeler transform ($BWT$) of $T$ is a permutation of $T$ derived from $SA_T$. Given $SA_T$, the $BWT$ of $T$, denoted by $B_T$, is obtained by the formula $B_T[i] = T[SA_T[i] - 1]$ for $SA_T[i] > 1$; and $B_T[0] = \$$. $BWT$ is highly compressible and it can be stored using $nH_k + o(n)$-bit space. $BWT$ is a method suitable for indexing large genomes. It requires around 750MB for a genome as large as hg19.

With the help of some auxiliary data structures, $BWT$ enables us to compute the SA range of a pattern using backward search.

**Lemma 1.1 (Backward Search)** *Consider a genome $T$. Given the BWT $B_T$ and an auxiliary data structure of size $o(n \log n)$ bits. Let $[i, j]$ be the $SA_T$ range of $P$. Then, the $SA_T$ range of $yP$ can be computed in constant time.*

**Proof** Let $C(y)$ be the total number of characters in string $T$ that are lexicographically smaller than $y$. $C(y)$ for all $y \in \Sigma$ can be stored in $O(\log n)$ bits.

Let $Occ_S$ be a function such that, for any $y \in \mathcal{A}$ and $1 \leq i \leq n$, $Occ_T(y, i)$ is the number of occurrences of $y$ in $B_T[1..i]$ and $Occ(y, 0) = 0$. As shown in [**?**],

we can store the $Occ_T$ data-structure using $o(n \log n)$-bit space while allowing constant time access to any entry $Occ_T(y, i)$.

If $[i, j]$ is the $SA_T$ range of $P$, [?] showed that the $SA_T$ range of $yP$ is $[C(y) + Occ_T(y, i1) + 1, C(y) + Occ_T(y, j)]$. Since the functions $C()$ and $Occ_T()$ can be computed in $O(1)$ time, the lemma follows. ∎

Furthermore, the following useful lemma can be shown to be true.

**Lemma 1.2 (BWT Inversion)** *Given the permutation of* $T[i]$ *under the* $BWT$*, and the auxiliary data structures in lemma 1.1, the permutation of* $T[i+1]$ *under the* $BWT$ *can be obtained in* $O(1)$ *time.*

We can find the $SA_T$ range of any pattern $P$ by starting off with the empty string, whose $SA_T$ range is $[1, n]$, and searching backward the characters of $P$ using Lemma 1.1. This is called the backward search for $P$ in $T$. If $P$ does not exist in $T$, the result of the backward search is not a valid interval.

If the $SA_T$ range of $P$ is a valid interval $[i, j]$, the following lemma enables us to retrieve $SA_T[k]$ for each $k = i, \ldots, j$ in $O((j - i + 1)\kappa)$ time.

**Lemma 1.3** *Given an* $O((n \log n)/\kappa)$*-bit auxiliary data-structure, for any* $k$*,* $SA_T[k]$ *can be computed in* $O(\kappa)$ *time.*

**Proof** As a pre-processing step, we sample and store each $SA_T[i]$, where $i$ is a multiple of some constant integer $\kappa$. We can compute an arbitrary $SA_T[k]$ using this sampling and $B_T$. If $k$ is a multiple of $\kappa$, $SA_T[k]$ can be found from the sampling. Otherwise, starting at $l = k$, by repeatedly applying Lemma 1.2 we count the number of steps $s'$ needed to arrive at the first point where $l'$ is a multiple of $\kappa$. Then $SA_T[k]$ is given by $SA_T[s] - s'$. This procedure needs on average $\kappa/2$ applications of the lemma. ∎

We choose $\kappa = O(\log n)$ in practice. In summary, using the BWT of $T$, we can store the index using $nH_k + o(n \log n)$ bits and enable us to find all hits of a pattern $P$ using $O(|P| + occ \log n)$ time where $occ$ is the number of hits of $P$ in $T$.

If an $SA_T$-range $[i, j]$ is given, we can find all locations corresponding to it by simply using Lemma 1.3 to calculate $SA_T[k]$ for $i \leq l \leq j$. However, this basic method of converting $SA_T$ ranges to locations can be time consuming, and we will next describe how to improve this step.

First we will describe how to efficiently convert an $SA_T$-value to the corresponding genomic location on the fly while performing a backward search with the following lemma.

**Lemma 1.4** *Let* $P$ *be a substring of* $T$*. When performing backward search for* $P$*, if the* $SA_T$*-range corresponding to* $P[1..l]$ *is in the form* $[\kappa i, \kappa i]$*, Then* $P$ *occurs uniquely at the location* $SA[\kappa i] - (|P| - l)$*.*

Since $SA_T[\kappa i]$ is already sampled, if any backward search satisfies this condition, we can obtain the location of $P$ in constant time as soon as the search terminates. For sufficiently long $P$, we can expect $P$ to occur uniquely in $T$ and to satisfy the above condition.

## 1.3 Retrieving Hits for a Fixed Length Pattern

Although BWT is space-efficient and can find the exact hits of a pattern, we need an average of $O(\log n)$ time to retrieve each hit. When the list of hits is long, it is time consuming to retrieve all hits.

We usually need to find hits of patterns having some fixed length $l$ when mapping reads. Below, we describe a simple data-structure that will speedup the retrieval of such hits.

The basic observation is that, when $l$ is long (say, $> \log_{|\Sigma|} n$), the $SA_T$-range for a length-$l$ pattern is usually short. In fact, assuming the genome sequence is a random string, we can show that the expected size of the $SA_T$-range for a length-($\log_{|\Sigma|} n$) pattern is 1. If we don't store $SA_T$ values for patterns whose $SA_T$-ranges are short, the data-structure will be small enough.

On the other hand, if the $SA_T$-range of a length $l$ pattern is too long, the pattern is more likely to be extracted from low complexity or repeat regions. Since such patterns tend to be noisy, we will not use those hits.

Based on these two observations, our data-structure stores the $SA_T$ values for patterns whose $SA_T$-ranges are of size between $\delta$ and $D$ ($\delta < D$) as follows. Let $\mathcal{P} = \{P_1, \ldots, P_r\}$ be the set of length-$l$ substrings of $T$ whose number of hits in $T$ are between $\delta$ and $D$. Let $[s_k, e_k]$ be the $SA_T$-ranges of $P_k$ for $k = 1, 2, \ldots, r$. Note that the $SA_T$-ranges of all these patterns do not overlap. We define the reduced suffix array $RSA_T$ to be an integer array formed by retaining only the $SA_T$ values in $[s_k, e_k]$ for all $k = 1, \ldots, r$. $SA_T$ values belonging to each $[s_k, e_k]$ are kept sorted in their positional order. Furthermore, we also store two arrays $S[1..r]$ and $SUM[1..r]$ where $S[k] = s_k$ and $SUM[k] = \sum_{i=1}^{k-1}(e_i - s_i + 1) + 1$. We denote our data-structure as $L_{T,l,\delta,D}$, and it consists of the $BWT$ of $T$, $RSA_T$, $S$ and $SUM$.

Figure 1 shows an example. Consider the string "$T = cccctgcggggccg\$$". The string contains 2-mers $cc, cg, ct, gc, gg$ and $tg$. The $BWT$ of $T$ is "$tccgccgc\$gggccg$". Only the 2-mers $cc, gg$ and $cg$ appear more than once in $T$. Figure 1(b) lists down the occurrences of each of these in $T$ sorted by their location, along with the $SA$-ranges. To construct $L_{T,2,2,10}$, we need to consider all 2-mers that appear at least twice and at most ten times, and the required data can be found in Figure 1(c). In the Figure, the $SA$-ranges are sorted by their starting position. Then, these starting positions are used as keys to the list of sorted locations belonging to the corresponding $SA$-ranges. If we are to construct $L_{T,2,2,3}$, then the entry for $cc$ will be removed as it contains more than 3 entries. Table 1 shows that $L_{T,l,\delta,D}$ is space-efficient in practice, for a large genome like hg19 and for $\delta = 4$ and $D = 30,000$.

Now, given $L_{T,l,\delta,D}$ and the $SA_T$-range $[i, j]$ of a pattern $P$ of length $l$, we can use algorithm $SA\_to\_Loc$ in Figure 2 to get all of its occurrences in $T$ efficiently. If $P$ occurs at most $\delta$ times, or more than $D$ times, we use the method given in Lemma 1.3 to obtain each $SA_T[i], \ldots, SA_T[j]$. Otherwise, we use $L_{T,l,\delta,D}$ to find all occurrences.

| $l$-mer size | Size of $L_{T,l,\delta,D}$ |
|---|---|
| 18 | 2.5 GB |
| 25 | 1.6 GB |
| 30 | 495 MB |
| 37 | 361 MB |
| 68 | 244 MB |
| 75 | 199 MB |
| 90 | 140 MB |
| 100 | 116 MB |

Table 1: Size of the data structure $L_{T,l,\delta,D}$ for different values of $l$, where $T$ is the hg19 genome, $\delta = 4$ and $D = 30,000$.

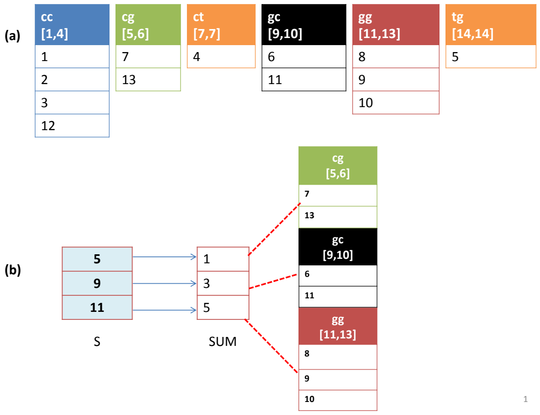


Figure 1: Illustration of the data structure for fast decoding of SA ranges for an example string $T = CCCCTGCGGGGCCG\$$. (a) shows the sorted positions of every non-unique 2-mers in the string. The label on top of of each list indicates the 2-mer and its SArange. (b) Data structure $L_{T,2,2,10}$ that indexes each 2-mer by the starting position of each SA-Range. The 2-mers $TG$ and $CT$ are not indexed as they occur uniquely in $T$. The 2-mers $CC$ and $GG$ do not appear as they appear more than twice. See Section 1.3 for details.

# 2 Bat-Align Mapping Strategy

Consider a genome $T$ and a read $R$. This section describes the mapping strategy of Bat-Align. Our strategy first finds a set of candidate mapping locations (called hits) of $R$ in $T$. The set of candidate hits is found by incrementally searching for the most likely combination of mismatches and indels. Based on the quality information and Smith-Waterman alignment, the best location is determined among them. Precisely, for every read $R$, our method performs three steps:

```
SA_to_Loc([i, j], L_{T,l,δ,D})

if (j − i) > δ and (j − i) ≤ D then
    Binary search S for the key i;
    if S[k] == i then
        return RSA[SUM[k]], ..., RSA[SUM[k + (j − i)] − 1];
    else
        return ∅;
    end if
else
    H = ∅;
    for k = i, .., j do
        L=location corresponding to k found by inverting BWT.
        H = H ∪ L
    end for
    return H;
end if
```

Figure 2: Algorithm $SA\_to\_Loc([i, j], L_{T,l,\delta,D})$ to convert $SA_T$-ranges to locations.

1. Finding candidate hits: This step identifies the hits of $R$ that minimize the number of mismatches and indels using two strategies Reverse alignment and Deep scan;
2. Rescore and select the best hit: This step uses the quality scores and Smith Waterman algorithm to rescore the hits and identify the best hit.
3. Calculate the mapQ score of the best hit and report it.

In the following subsections, we will describe these three steps.

## 2.1 Finding Candidate Hits

Given a read $R$, this step aims to incrementally find the hits of $R$ with the most likely combination of mismatches and indels. Its algorithm *Gen_Hits* is illustrated in Figure 3. we define a function $F$ such that $F(i) = (p_i, q_i)$, where $i, p_i$ and $q_i$ are non-negative integers representing the number of mismatches and indels in an alignment respectively.The algorithm *Gen_Hits* incrementally tries to map $R$ allowing $F(i)$ mismatch/indel combinations for $i = 0, 1.., M$. We call this method of incremental scanning using $F$ as Reverse alignment. If $F(k)$ is the first such successful mismatch/indel combination, and there are multiple hits, we return all these hits. If there is a unique hit and $k < M$, we return all hits having the mismatch/indel combinations $F(k)$ and $F(k + 1)$. We call this step the Deep scanning step.

In real-life, the likelihood of an indel in a genome is an order of magnitude less than that of a SNP and the likelihood of finding multiple indels becomes

```
Gen_Hits(R, G, F, M)

for i = 0 to M do
    H1=Hits of R in G having F(i) mismatch/indel combinations.
    if H1 is not empty then
        if H1 contains unique hit and i < M then
            H2=Hits of R having F(i + 1) mismatch/indel combinations.
            return H1 ∪ H2.
        else
            return H1
        end if
    end if
end for
```

Figure 3: Algorithm *Gen_Hits* to generate a candidate set of hits for examination

small as the length of $R$ becomes small. If the sequencing is ideal and without any errors, we can expect mismatches in the read $R$ to appear at a rate equal to the expected number of SNPs in a segment of length $|R|$ in $G$. However, empirical studies show that, for Illumina and SOLiD, the majority of mismatch errors occur due to sequencing errors. A general heuristic for such platforms is to set the mismatches in a read due to sequencing errors and SNPs to be about 4-5% of the read length. Furthermore, indels occur at a frequency of around 1 out 10k bases. Based on these figures, for a read of length around 75bp, we can set one indel and four mismatches as a reasonable upper bound for the number of indels and mismatches to be allowed in a mapping. For the default mode of enumerating candidate hits, We set $F^{-1}(n_1, n_2) < F^{-1}(n_1 + 1, n_2)$ for $n_1 \leq 4$ and $F^{-1}(5, 0) < F^{-1}(0, 1)$.

We use efficient BWT-based methods to enumerate hits corresponding to $F(i)$. With the restriction stated above, i.e. assuming we allow only one indel, we have two cases where $F(i)$ is of the form $(p_i, 0)$ or $(p_i, 1)$. When $F(i) = (p_i, 0)$, i.e. when only mismatches are allowed in $R$, we use the algorithm *BatMis* [**?**]. *BatMis* is a BWT-based algorithm that can be used to solve the $k$-mismatch problem efficiently. The following section describes the algorithm used to handle the case of a read having one indel in detail.

### 2.1.1 Finding Indel Hits.

When $F(i) = (p_i, 1)$, we allow $p_i$ mismatches along with an indel. The algorithm *Find_Indel* is described in Figure 4. To find these hits, there are two cases. The first case is that the indel appears in one half of the read. Then the half of the read that does not contain the indel must contain at most $p_i$ mismatches only. We identify hits in $T$ having at most $p_i$ mismatches with one half of $R$ as

$Find\_Indel(R, G, p_i)$
{/* Case 1: Indel appears in one half of the read */}
$X_1$= The set of $p_i$ mismatch hits of $R[1..|R|/2]$ in $G$.
$H_1$= The set of hits having $p_i$ mismatches and one indel obtained by Smith-Waterman extension of hits in $X_1$.
$X_2$= The set of $p_i$ mismatch hits of $R[|R|/2+1..|R|]$ in $G$.
$H_2$= The set of hits having $p_i$ mismatches and one indel obtained by Smith-Waterman extension of hits in $X_2$.
{/* Case 2: Indel is not completely contain in either half of the read */}
**for** $j = 0$ to $p_i$ **do**
    $X_j$= The set of hits of $R[1..l]$ in $G$ having $j$ mismatches.
    $Y_j$= The set of hits of $R[|R|-l+1..|R|]$ in $G$ having $j$ mismatches.
**end for**
**for** $j = 0$ to $p_i$ **do**
    $H = H \cup \{$ Hits in $X_j$ and $Y_{p_i-j}$ that are within $|R|-2l+d$ bases from each other.$\}$
**end for**
$Hits$ = The set of hits in $H$ that can be extended to have $p_i$ mismatches and one indel.
**return** $H_1 \cup H_2 \cup Hits$.

Figure 4: Algorithm $Find\_Indel$ to find hits having one indel.

seeds and perform Smith-Waterman extension to recover locations having $p_i$ mismatches and one indel for the whole read $R$.

The second case is that the indel is not completely contained in either half but is in the middle of the read, $Find\_Indel2$ is used. It will find $0, .., p_i$ mismatch hits of each $l$-mer suffix and prefix of $R$ using the $BatMis$ algorithm. Then it will find the suffix and prefix locations that are at most $|R|-2l+d$ bases apart using the method described in Section 2.1.2. Here, the value for $l$ should be determined based on the maximum size of an insertion that is allowed. These potential hit locations are further examined by aligning against $R$ with the Smith-Waterman algorithm, and those hits with $p_i$ mismatches and one indel are reported.

### 2.1.2 Efficiently Pairing Two Patterns.

Given two patterns $P_1$ and $P_2$ of length $l$, we say that they can be paired if the two patterns can be found within a distance $d$ of each other, with $P_1$ having the least genomic co-ordinate. Let $Occ_T(P)$ denote the number of times the pattern $P$ occurs in $T$. We will describe now how to efficiently find pairings of $P_1$ and $P_2$ when $L_{T,l,\delta,D}$ is given and when $Occ_T(P_1) < D$ and $Occ_T(P_2) < D$.

1. If $Occ_T(P_1) < \delta$ and $Occ_T(P_2) < \delta$, we find all locations of $P_1$ and $P_2$. Then these locations can be cross checked to obtain all pairings of $P_1$ and $P_2$.

2. If $Occ_T(P_1) < \delta$ and $Occ_T(P_2) \geq \delta$, we obtain all the locations of $P_2$ from $L_{T,l,\delta,D}$. Since these are sorted by their position, we can check to see if any occurrence of $P_1$ can be paired with an occurrence of $P_2$ by performing a binary search.

3. If $Occ_T(P_1) \geq \delta$ and $Occ_T(P_2) < \delta$ , the above procedure can be modified by looking up all the occurrences of $P_1$ from $L_{T,l,\delta,D}$.

4. If $Occ_T(P_1) \geq \delta$ and $Occ_T(P_2) \geq \delta$, all the occurrences of $P_1$ and $P_2$ are obtained from $L_{T,l,\delta,D}$. These two lists are merge sorted. If an occurrence of $P_1$ is followed by an occurrence of $P_2$, and they satisfy the distance constraint, we report them.

## 2.2 Reporting Hit.

Sequencing data usually produces a quality score that indicates the reliability of a base call. If the probability of a base call at position $i$ being correct is $P(i)$, the quality score $Q(i)$ assigned to location $i$ is given by the equation $P(i) = 1 - 10^{-Q(i)/10}$. Assuming that there is no bias to a particular set of nucleotides when a base gets miscalled with another nucleotide, then the probability of a base being miscalled at location $i$ can be calculated by the formula $1 - P(i)/3$. For a given alignment, we compute an alignment score based on an affine scoring scheme. The score for a match or a mismatch at $R[i]$ is the phred scaled value of $P(i)$. When indels are present, positive gap open and extend values are chosen.

From Section 2.1, a candidate set $X$ of mapping locations can be obtained for each read. Next, for each alignment in $X$ we calculate the alignment score. If a unique alignment exists, it is reported. Otherwise, if the difference of alignment scores between the two smallest alignment scores is larger than some suitable cutoff(set as 10), the alignment with the smallest score is reported.

### 2.2.1 Increasing Sensitivity and Accuracy.

In Section 2.1 we described our basic criteria for finding a suitable set of hits. This criteria will miss enumerating the correct hit under three cases.(1) The read contains mismatches and indels outside the scope the algorithm is set to handle. (2) The read contains only $m$ mismatches, but have a multiple mapping having less than $m$ mismatches or a unique mapping having less than $m - 1$ mismatches.(3) A read containing indels with $m$ mismatches might get mapped as pure mismatch hit or as an indel hit having less than $m$ mismatches. We use several methods to recover these three kinds of hits.

During the stage described in Section 2.1.1, we might recover possible hits having more than the number of mismatches and indels specified by the basic algorithm. Instead of discarding them we will add them to the pool of candidate hits. If a hit found in the mismatch scanning stage turns out to have an indel when the full-read extension is done, an indel scan is forced. If the best alignment score happens to be larger than that of a single indel hit, again an

indel scan is forced. Finally, for reads having more than one mismatch, the indel scanning is performed if the average quality of bases having a mismatch is higher than that of the average base quality of the read. We allow one high quality mismatch to consider the possibility of a SNP.

#### 2.2.2 Making the Algorithms Faster.

We can use some strategies to speed-up the algorithm. These will result in a loss of accuracy and sensitivity. When extending the candidate hits along the full read at the indel stage. Instead of extending all the hits, we terminate the extension process, if for $K$ successive steps, the extension has not resulted in an improvement in the alignment score. Furthermore, the extension can be limited to those hits already having an indel when an indel scan is forced.

The methods in Sections 2.2.1 and 2.2.2 has been used in various combinations to produce several modes of scanning that trades speed with accuracy and sensitivity.

### 2.3 Calculating Mapping Quality

The mapping quality is calculated as a measure to indicate the accuracy of the alignment, ranging from 0 (indicate a mapping without any confidence) to a value between 1 and 60, indicating the lowest and highest confidences for a hit that can be reported unambiguously. The mapping quality takes into account factors including the sequencing quality, the likelihood of the alignment appearing in the reference genome and the likelihood of the hit appearing by chance. We give an affine gap score $S_{ref}$ to the final alignment to measure the likelihood of the sequence appearing in the reference. During the alignment stage, we count how many alignments $S_{multi}$ the first half of the read will have when 0 mismatches are allowed. A large value for $S_{multi}$ indicates a higher chance that the read will align by merely by chance. The final score $S_{prior}$ uses quality information to factor in the prior probability of the read aligning at the reported location as explained below.

Let $A_{top}$ be the best alignment returned by the aligner. Then we can calculate the prior probability to be

$$p(A_{top}|G,R) = \frac{p(R|G,A_{top})}{\sum_{i=1}^{|G|} p(R|G,x)},$$

where $p(R|G,A_j)$ is the chance of $R$ aligning at position $A_{top}$. A higher value of $p(R|G,A_{top})$ indicates a higher prior probability. For mismatch hits, $p(R|G,A_j) = 10^{-(\sum_i Q_i)/10}$, where $Q_i$ are the quality scores at the mismatched bases. We set $S_{prior} = p(R|G,A_{top})$.

The final mapping score is calculated by the formula $60 - k_1 S_{ref} - k_2 \min(10, S_{multi}) + k_3 S_{prior}$, where $k_1, k_2$ and $k_3$ are three positive constants that are empirically determined.

## 3. Evaluation on simulated reads

### 3.1 ART-simulated reads

We have simulated three dataset to evaluate the performance of the various methods in the main article. The mappings were stratified accordingly to the mapQ that the corresponding program assigns to them. The results were plotted with cumulative number of alignments, in order of decreasing mapQ, and were used to plot Supplementary Figure 3.1.

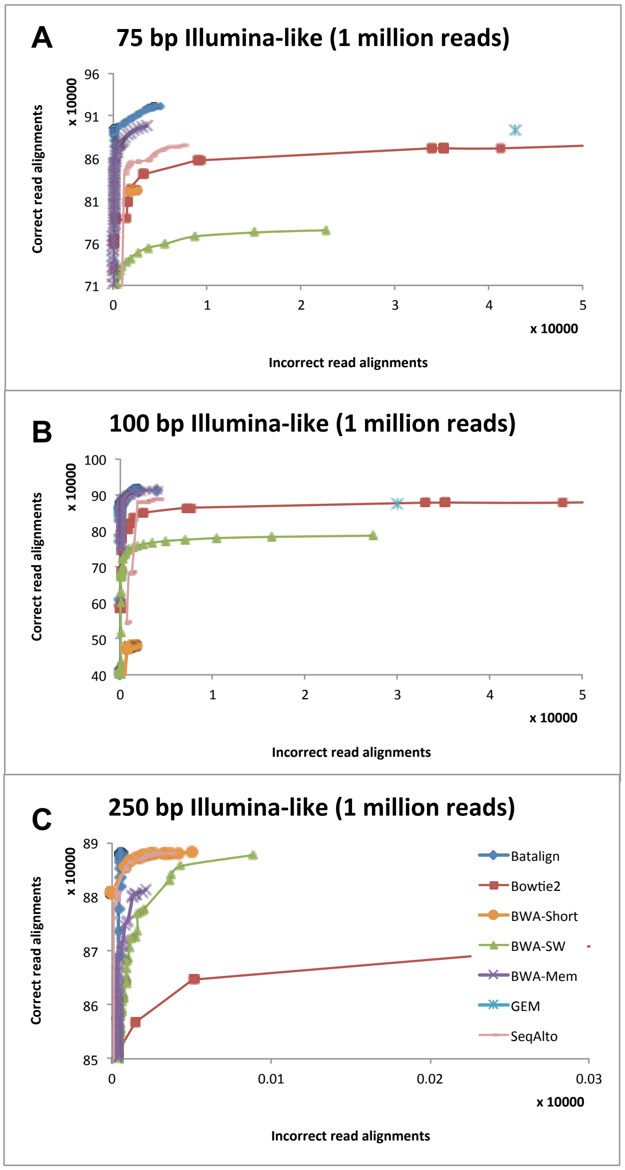


Supplementary Figure 3.1. Cumulative alignments stratified by decreasing mapQ as reported by their respective aligners in the form of ROC graphs. S.Figure A/B/C for 75/100/250 bp reads.

Similar to the experiments performed in GEM's paper, we also validated the top 10 hits reported by each method. The complete breakdown of this validation by the first (or best) alignment, as ordered by their respective aligner, can be found in Supplementary Table 3.1. From this table, we can see that BatAlign has reported the most number of correct hits as top-ranked hits across the 3 datasets of various read-lengths. It was only for the total correct hits on the 250 bp dataset that BatAlign is second to GEM by a small margin of < 0.01%. Upon inspection on the extra reads which GEM has reported, BatAlign found them to be repetitive and it was by chance that these hits were not represented as any of the top 10 hits for these reads.

Supplementary Table 3.1. Number of correct hits ordered by the rank that each aligner reported.

| Rank / Aligner | 75bp dataset #Correct hits 1 | 2 | 3 | 4 | 5 | 6 | 7 | 8 | 9 | 10 | Sum of correct hits |
|---|---|---|---|---|---|---|---|---|---|---|---|
| BatAlign | 933560 | 11604 | 732 | 622 | 745 | 335 | 146 | 116 | 90 | 68 | 948018 |
| Bowtie2 | 866410 | 10873 | 4095 | 1541 | 551 | 314 | 192 | 142 | 112 | 77 | 884307 |
| BWA-SW | 786309 | 2 | 0 | 0 | 0 | 0 | 0 | 0 | 0 | 0 | 786311 |
| BWA-Mem | 897581 | - | - | - | - | - | - | - | - | - | 897581 |
| GEM | 893162 | 13603 | 5243 | 2231 | 1074 | 688 | 555 | 375 | 323 | 272 | 917526 |
| BWA-Short | 834519 | 10008 | 3535 | 1226 | 408 | 160 | 83 | 52 | 43 | 26 | 850060 |
| Seqalto | 885208 | 5692 | 1712 | 723 | 306 | 164 | 102 | 63 | 33 | 32 | 894035 |

| Rank / Aligner | 100bp dataset #Correct hits 1 | 2 | 3 | 4 | 5 | 6 | 7 | 8 | 9 | 10 | Sum of correct hits |
|---|---|---|---|---|---|---|---|---|---|---|---|
| BatAlign | 924272 | 7599 | 941 | 728 | 851 | 332 | 182 | 108 | 101 | 63 | 935177 |
| Bowtie2 | 866310 | 8685 | 2833 | 948 | 254 | 110 | 69 | 42 | 23 | 5 | 879279 |
| BWA-SW | 794661 | 7 | 0 | 0 | 0 | 0 | 0 | 0 | 0 | 0 | 794668 |
| BWA-Mem | 912562 | - | - | - | - | - | - | - | - | - | 912562 |
| GEM | 875333 | 10327 | 3533 | 1445 | 638 | 377 | 283 | 193 | 163 | 178 | 892470 |
| BWA-Short | 484558 | 5207 | 1747 | 662 | 211 | 112 | 79 | 48 | 20 | 13 | 492657 |
| Seqalto | 890336 | 1821 | 515 | 194 | 91 | 44 | 38 | 11 | 3 | 8 | 893061 |

| Rank / Aligner | 250bp dataset #Correct hits 1 | 2 | 3 | 4 | 5 | 6 | 7 | 8 | 9 | 10 | Sum of correct hits |
|---|---|---|---|---|---|---|---|---|---|---|---|
| BatAlign | 894799 | 6394 | 732 | 824 | 225 | 175 | 107 | 98 | 53 | 50 | 903457 |
| Bowtie2 | 892350 | 6245 | 1269 | 346 | 184 | 131 | 83 | 62 | 49 | 34 | 900753 |
| BWA-SW | 894395 | 1 | 0 | 0 | 0 | 0 | 0 | 0 | 0 | 0 | 894396 |
| BWA-Mem | 881193 | - | - | - |  | - | - | - | - | - | 881193 |
| GEM | 895450 | 5999 | 1203 | 319 | 201 | 116 | 92 | 79 | 50 | 46 | 903555 |
| BWA-Short | 894658 | 5615 | 800 | 67 | 0 | 0 | 0 | 0 | 0 | 0 | 901140 |
| Seqalto | 894661 | 3775 | 296 | 77 | 41 | 33 | 14 | 9 | 9 | 9 | 898924 |

### 3.2 Simulated Indel-abberrant reads

We have also simulated a new read class – Indel-abberrant datasets for analyzing the performance of the various programs on mapping indel-aberrant reads. We have tested various programs on read set with indel ranging from 1bp to 8bp long; each with 1 million 75 bp reads. For each of the indel-lengths, we further classified the simulated-variant into insertions or deletion class. Supplementary Table 3.2 detailed on the calculated F-measures in the main paper.

Supplementary Table 3.2. Results on mapping Indel-abberrant datasets by various programs

| Methods | Delete-aberrant | | |
|---|---|---|---|
| | Correct hits | Wrong hits | F-measure |
| BatAlign | 856069 | 5365 | 92.0% |
| Bowtie2 | 819007 | 70844 | 86.7% |
| BWA-Short | 764699 | 2580 | 86.5% |
| BWA-SW | 719140 | 22798 | 82.6% |
| GEM | 833640 | 38252 | 89.1% |
| SeqAlto | 813325 | 7593 | 89.3% |
| BWA-Mem | 833994 | 3746 | 90.8% |

| Methods | Insert-aberrant | | |
|---|---|---|---|
| | Correct hits | Wrong hits | F-measure |
| BatAlign | 855681 | 5586 | 91.9% |
| Bowtie2 | 818760 | 70977 | 86.7% |
| BWA-Short | 764275 | 2674 | 86.5% |
| BWA-SW | 718737 | 23051 | 82.5% |
| GEM | 833295 | 38395 | 89.0% |
| SeqAlto | 812779 | 7526 | 89.3% |
| BWA-Mem | 833396 | 3891 | 90.7% |

### 3.3 Reads from a RSVsim rearranged genome

WGSIM was used to simulate 2x5Mx100bp reads from hg19-chr21 for the analysis of alignment-performance of the compared methods in this section. Reads were simulated with the default of 2% sequencing errors and 1% polymorphism and aligned back to the original UCSC hg19 reference genome. Variants are called using Samtools and Vcftools.

We realized that different affine gap penalty scores used by different aligners could result in different clipping lengths of the reads in their corresponding alignments. Clipping will omit possible coverage above sites of polymorphic variants even when their alignments correct reflect the origin of the sequenced read. In order to minimise this bias, we only consider variants that are not within 50 bp of each other.

Below, we tabulated the F-measures of the called variants from the alignments of their respective aligners at various depth-cutoff.

Supplementary Table 3.3. F-measures of called SNVs and Indels from WGSIM-simulated reads at various depth-cutoff thresholds.

| Methods | F-measures of SNV-calling (at various depth-cutoff) | | |
|---|---|---|---|
| | 2 | 5 | 10 |
| BatAlign | **99.4%** | **99.4%** | **99.3%** |
| Bowtie2 | 98.0% | 98.4% | 96.0% |
| BWA-Short | 99.0% | 99.0% | 98.9% |
| BWA-SW | 98.5% | 98.5% | 98.3% |
| GEM | 96.6% | 95.6% | 94.3% |
| SeqAlto | 99.0% | 99.0% | 98.9% |
| BWA-Mem | 99.1% | 99.1% | 99.0% |

| Methods | F-measures of Indel-calling (at various depth-cutoff) | | |
|---|---|---|---|
| | 2 | 5 | 10 |
| BatAlign | **86.3%** | **86.3%** | 86.2% |
| Bowtie2 | 74.9% | 77.4% | 73.5% |
| BWA-Short | 84.6% | 84.7% | 84.2% |
| BWA-SW | 85.0% | 85.0% | 84.9% |
| GEM | 62.4% | 62.2% | 61.6% |
| SeqAlto | 84.2% | 84.2% | 84.1% |
| BWA-Mem | **86.3%** | **86.3%** | **86.3%** |

# 4 Evaluation on real reads

### 4.1 1 million reads data sets

We have downloaded three dataset, DRR000614, SRR315803 and SRR850313 to evaluate the performance of the various methods in the main article. From each of the dataset, 1 million read were extracted and mapped by the various stated programs. The mappings were stratified accordingly to the mapQ which the corresponding program assigned to them. The results were plotted with cumulative number of alignments, in order of decreasing mapQ of the 'head' alignment, in Supplementary Figure 4.1.

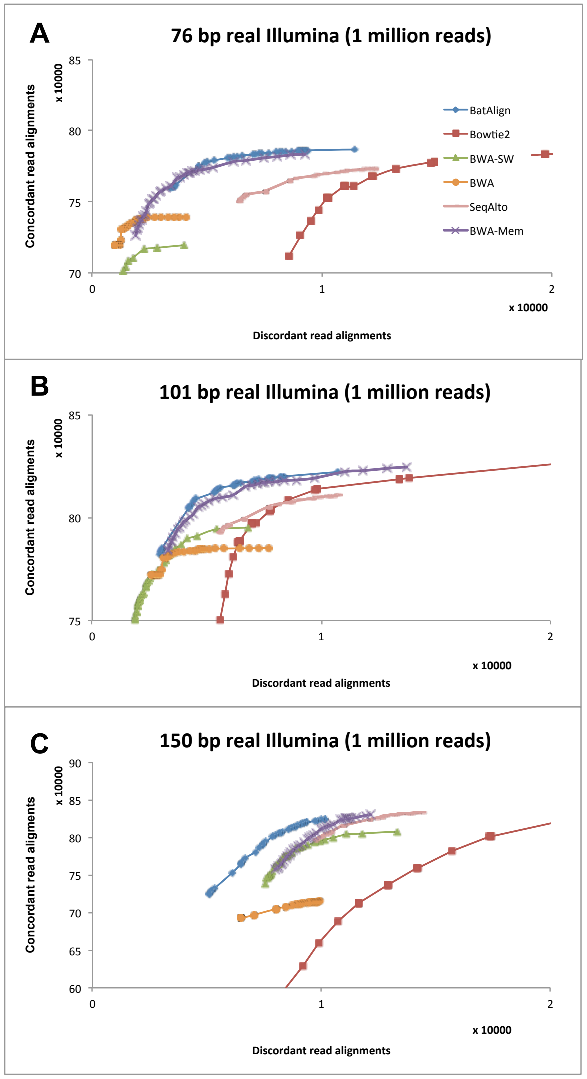


Supplementary Figure 4.1. Cumulative concordant & discordant alignments stratified in order of decreasing mapQ as reported by their respective aligners in the form of ROC graphs.

### 4.2 Evaluation on life-sized data

For real WGS data, we have only used the primary, non-secondary alignment reported for each read from the compared methods. On WGS data, we realized that BreakDancer might miss an SV if read_1 is a breakpoint-spanning read. In this case, either the primary or the supplementary alignment will form a concordant read-pair alignment with read_2 and can elude the discovery of a structural variant. 800 bp insert size from ERP001196's 11T, de-multiplexed from 4 lanes, were used as the discovery set for SNV-callings. Results of the callings, with variant scores >=30, were tabulated in Supplementary Table 4.1 and 4.2.

Supplementary Table 4.1. Comparison on the number of SNVs recalled from published and validated SNVs of Patient 11T at various depth cutoff. Best results are in bold.

| Methods | Intersect with published 11T data (with different depth-cutoff) | | |
|---|---|---|---|
| | 2 | 5 | 10 |
| BatAlign | **45** | **42** | **37** |
| Bowtie2 | 0 | 0 | 0 |
| BWA | 42 | 40 | 35 |
| BWA-SW | 44 | 41 | 36 |
| GEM | 43 | 41 | 35 |
| SeqAlto | 1 | 1 | 0 |
| BWA-Mem | **45** | **42** | **37** |

Supplementary Table 4.2. Total number of SNVs called from data of Patient 11T at various depth-ctRutoff.

| Methods | Total SNVs called at various depth cutoff | | |
|---|---|---|---|
| | 2 | 5 | 10 |
| BatAlign | 2984901 | 2839346 | 2207313 |
| Bowtie2 | 3003669 | 2506634 | 1098618 |
| BWA | 3052128 | 2939975 | 2372334 |
| BWA-SW | 2798359 | 2668373 | 2063522 |
| GEM | 4015855 | 3837328 | 3075882 |
| SeqAlto | 237425 | 172190 | 123160 |
| BWA-Mem | 3159183 | 3029818 | 2439042 |

The library used had ~16X in sequencing depth. A total of 80 validated SVs were [27] used for this comparison.

## 5 Parameters used for all compared programs

### Single-end Read Settings

Bat-Align (Default)
./penguin –g <INDEX> -q <INPUT > -o <OUTPUT>

Bat-Align (Fast)
./penguin –g <INDEX> -q <INPUT > -o <OUTPUT> --mode=vfast --swlimit=10

Bat-Align (Turbo)
./penguin –g <INDEX> -q <INPUT > -o <OUTPUT> --boost=5 --mode= vfast --swlimit=10

Bowtie2
./bowtie2 --local -x <INDEX> -U <INPUT > -S <OUTPUT>

BWA-SW
./bwa bwasw –f <OUTPUT> <INDEX> <INPUT>

GEM
./gem-mapper –q offset-33 –I <INDEX> -i <INPUT> -o <OUTPUT.gem>
./gem-2-sam –I <INDEX> -i <INPUT.gem> -o <OUTPUT> -q offset-33

BWA-short
./bwa aln <INDEX> <INPUT> > <SAI>
./bwa samse –f <OUTPUT> <INDEX> <SAI> <INPUT>

SeqAlto
./seqalto_basic align <INDEX> -1 <INPUT> > <OUTPUT>

BWA-Mem
./bwa mem -M <INDEX> <INPUT>

To investigate best (or first) correct alignments of the various methods, we had to input additional options for the programs. We listed the options for the programs which needed it to report 10 multi-hits in its output below. This was used in the comparisons of multi-hits in *Evaluation on ART-simulated reads* of the main paper.

Bat-Align used "--tophits 10". Bowtie2 used "-k 10". BWA-short used "-n 10". BWA-Mem used "-a". The other programs prints multi-hits by default and the number, which they can report, cannot be restricted manually by parameter-input.

### Paired-end mapping

Bat-Align (Default)
./penguin –g <INDEX> -q <INPUT1 > -q <INPUT2 > -o <OUTPUT>

Bowtie2

```
./bowtie2 --local -x <INDEX> -1 <INPUT1> -2 <INPUT2>  -S <OUTPUT>
```

BWA-SW

```
./bwa bwasw –f <OUTPUT> <INDEX> <INPUT1> <INPUT2>
```

GEM

```
./gem-mapper –q offset-33 –I <INDEX> -1 <INPUT1> -2 <INPUT2> -o <OUTPUTtmp> -p
./gem-2-sam –I <INDEX> -i <OUTPUTtmp>.gem -o <OUTPUT> -q offset-33
```

BWA-short

```
./bwa aln <INDEX> <INPUT1>  >  <SAI1>
./bwa aln <INDEX> <INPUT2>  >  <SAI2>
./bwa sampe –f <OUTPUT> <INDEX> <SAI1> <SAI2> <INPUT1> <INPUT2>
```

SeqAlto

```
./seqalto_basic align <INDEX> -1 <INPUT1> -2 <INPUT2> >  <OUTPUT>
```

BWA-Mem

```
./bwa mem -M<INDEX> <INPUT1> <INPUT2>
```

## 6 Evaluation on running time

### 6.1 ROC curves of Bat-Align (different modes)

We tabulated the mapping runtimes from all the programs Table 5 of the main article. The various modes of Bat-Align have attained a better ROC curves as shown below. All the discussed modes of Bat-Align in the main article have also achieved competitive running times as compared to the other programs.

Supplementary Figure 6.1. ROC curves plotted from different modes of Bat-Align and other methods on 1 millions reads extracted from SRR315803.

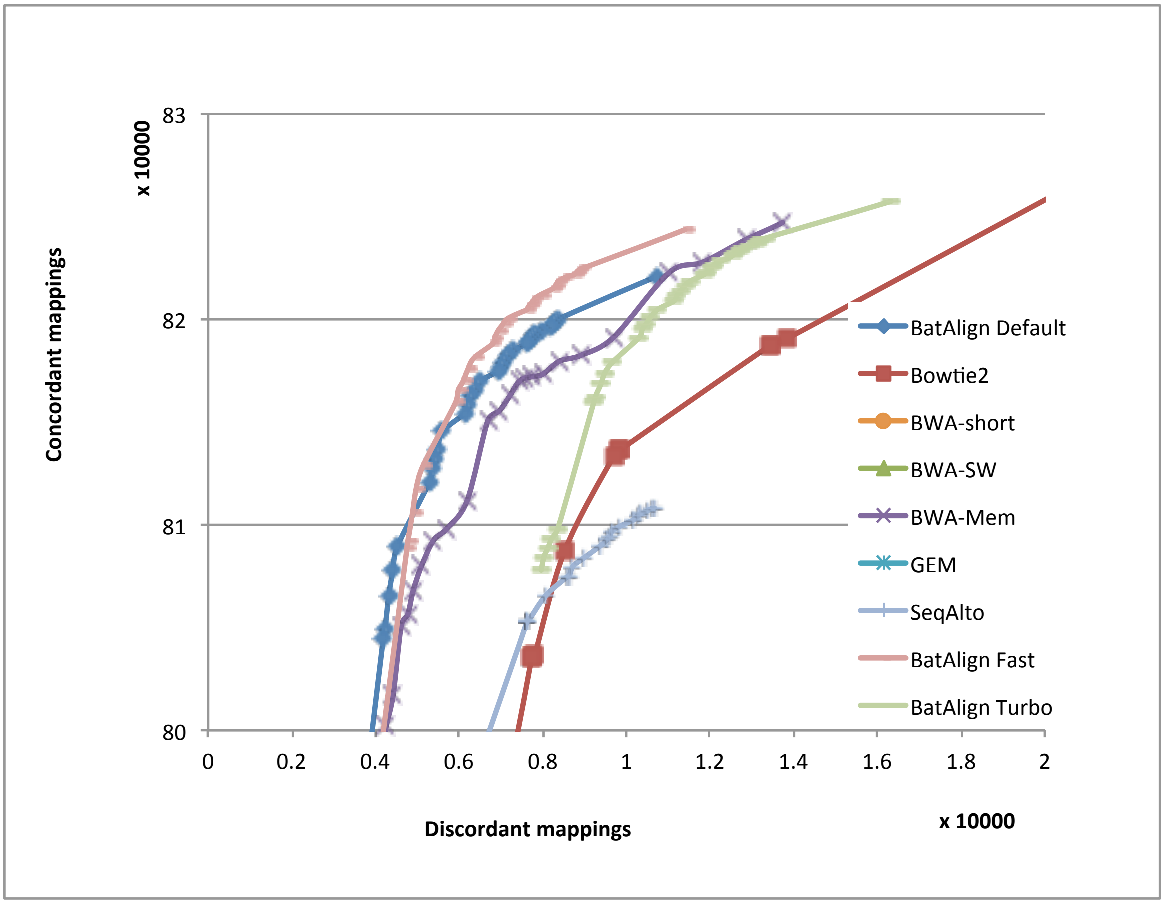

Supplement: SUPPLEMENTARY DATA [file supp_gkv533_nar-00379-met-n-2015-File008.pdf]
